# Spatial and socioeconomic patterns of COVID-19 in transition zones between municipalities in eastern Amazonia

**DOI:** 10.3389/fpubh.2025.1526642

**Published:** 2025-03-13

**Authors:** Dinar Duarte Vasconcelos, Hermes Fonsêca de Medeiros, José Antonio Herrera, Lucas de Oliveira Lima, Pedro Fernando da Costa Vasconcelos, Juarez Antonio Simões Quaresma

**Affiliations:** ^1^State University of Pará, Belém, Brazil; ^2^Postgraduate Program in Parasite Biology in the Amazon (BPA), Belém, Brazil; ^3^Department of Community Health, State University of Pará, Altamira, Brazil; ^4^Laboratory for Studies of Territorial Dynamics in the Amazon (Ledtam), Altamira, Brazil; ^5^Biology Faculty, Federal University of Pará, Altamira, Pará, Brazil; ^6^Federal University of Pará, Altamira, Pará, Brazil; ^7^Postgraduate Program in Geography (PPGEO), Altamira, Pará, Brazil; ^8^Department of Pathology, State University of Pará, Belém, Brazil; ^9^Postgraduate Program in Tropical Diseases, Tropical Medicine Center, Federal University of Pará, Belém, Brazil; ^10^School of Medicine, Federal University of São Paulo, São Paulo, Brazil

**Keywords:** SARS-CoV-2, COVID-19, spatial analysis, municipalities, Amazon biome, multivariate analysis, hospitalization, fatality

## Abstract

**Background:**

Herein, we assess hospitalizations and deaths from COVID-19 in Amazonian municipalities, taking into account regional, demographic, and socioeconomic peculiarities. Public data from 2020 and 2021 of 52,082 cases of COVID-19 were analyzed in R Program.

**Methods:**

We examined the interaction of mortality, hospitalization, and fatality rates of COVID-19, considering socioeconomic, demographic, and geographic variables. We measured the spatial autocorrelation of the rates associated with the variables POP, GDP, Residents, HDI, and GINI. The spatial patterns found show distinctly affected sectors and COVID-19 transition zones between municipalities.

**Results:**

We detected higher mortality rates in territories with greater social and environmental vulnerability. Analysis of the mortality rate indicates that all the socioeconomic variables tested are associated with this variable, but their effects interact in a complex way. The municipalities with higher numbers of residents per household and Gini coefficients had higher fatality rates, and municipalities with a higher GDP were more associated with higher hospitalization and mortality rates. Furthermore, the five socioeconomic indices included in multiple regressions analyzing mortality and hospitalization rates exhibited significant interaction effects. However, no significant interaction effects were observed in the fatality rate analyses.

**Conclusion:**

Spatial analyses showed that none of the 144 municipalities studied had high overlapping rates of mortality, hospitalization, and fatality rates for COVID-19 in the same municipality. We recommend further studies in the transition zones, considering the municipalities of *Floresta do Araguaia*, *Mãe do Rio*, and *Redenção* for mortality, *Barcarena, Capitão Poço*, and *Redenção* for hospitalization, and *Cumaru do Norte* and *Pau D’Arco* for fatality, in order to understand the health dynamics of each territory. The most affected areas are located near the border with the state of Amazonas. We recommend the adoption of personalized strategies for Amazonian municipalities when targeting future public health events.

## Graphical Abstract

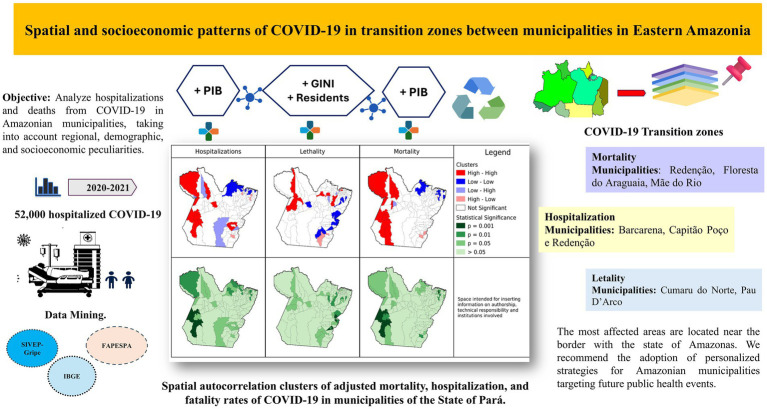

## Introduction

The SARS-CoV-2 virus, an etiological agent of COVID-19, of zoonotic origin, caused a global epidemic socioeconomic and public health impacts that vary according to geographic and time scales, and result in cascading effects that may persist for decades (1).

Several experts warned of a possible overload of the Brazilian health system, as the epidemic spread through capital cities and migrated to the interior, with a high virus contagion rate in any type of community environment, that made it more difficult to contain spatially (2, 3).

The Amazon presents important environmental and social characteristics that determine the epidemiological profile of infectious diseases, with implications for the health care network. These include geographic and ecological aspects, such as: the spatial scale of the phenomena, a large territorial extension, difficulty of access, which for certain areas occurs only by river, and rich biological, social, and cultural diversity, in addition to the degradation of natural ecosystems (4).

This area has complex ecosystems that are favorable to infectious diseases, due to social, environmental, territorial, and economic vulnerabilities and the lack of guarantees of fundamental rights to the population (5).

Located in the eastern Amazon, the State of Pará is divided into areas of greater regional integration, economic dynamics, and logistical aspects. It has regional health centers that are relatively independent in the implementation of public policy actions (6).

The described situation calls for inspection of “retrospective COVID-19” scenarios in the Amazon at different scales, regional particularities, and mapping of areas with higher and lower risks of hospitalization and deaths, since analysis of recorded epidemic patterns provides relevant information for the development of prevention policies and interventions, during and after health emergencies such as pandemics and environmental disasters. Projections of risk scenarios, knowledge of the territory, and the existing service capacity, highlight the differences in the “resilience of the health system” of the region (7). Territorial differences, human behaviors linked to local culture, and the spatial organization of administrative units for health care for the population are decisive in making assertive decisions and generating organically articulated actions in the Amazon territory (8).

The effects of environmental changes and the emergence of infectious diseases such as COVID-19 are a global warning that countries should adhere to a more collaborative agenda. In this sense, the One Health approach is recommended, which aims to find equitable, inclusive, and sustainable solutions in surveillance policies, with attention to the risks in the animal-human-environment relationship (9).

Global health governance is a challenge for managers in the face of adversities that make up the three domains of One Health (10), and the integration of surveillance networks, aligned with the sustainable development goals (SDGs), helps to meet the goals of the 2030 agenda.

In line with the pillars and main targets of SDG 3 (Good Health and Well-being), SDG 16 (Peace, Justice and Strong Institutions), and SDG 17 (Partnerships for the Goals) (11), our study provides scientific support for decision-making, improving operational capacity, and strengthening intersectoral actions in the surveillance network in the areas studied, as epidemiological research is a tool for applying One Health, presenting data and reporting fundamental scenarios for the biocontrol of infectious diseases.

In the Amazon, identifying spatial distances and territorial differences that imply critical delays in care, and defining local health priorities, involves observing changes in landscapes and patterns of zoonotic diseases in the territory, and strengthening the local health system, as such events disproportionately affect populations that require greater social protection, and are a challenge for public health. The Amazon region is considered challenging due to environmental degradation, socio-ecological vulnerability, and the risk of possible spillover (12, 13). The current diagnostic study favors the improvement of health surveillance measures, as, to date, spatial descriptions of COVID-19 associated with demographic and socioeconomic variables in Amazonian municipalities are scarce.

## Materials and methods

### Scope of the study

This is an ecological, cross-sectional, analytical, quantitative study carried out in 144 municipalities in the State of Pará, located in the northern region of Brazil. This area borders the states of Amazonas, Amapá, Mato Grosso, Tocantins, and Maranhão. In the geographical context, it is divided into 12 integration regions (Supplementary material), with 13 administrative units, known as regional health centers (14).

### Study population

A total of 76,406 cases of severe acute respiratory syndrome were reported in the state of Pará. The population of this study corresponded to all 52,082 confirmed hospitalized cases of COVID-19, between 2020 and 2021, according to protocols of the Brazilian Ministry of Health. It is noteworthy that the values of the population of this study corroborate those of the study carried out by Ranzani and cols (15).

### Data source

Data from official sources of the Influenza Epidemiological Surveillance System (SIVEP-Gripe) platforms were used, available to access in OpenDataSUS of Brazilian Ministry of Health, DATASUS (16), Brazilian Institute of Geography and Statistics (2010 Census) (17), and the Amazon Foundation for Support of Studies and Research (FAPESPA) (18), Mortality Information System (SIM), DATASUS (16), Human Development Atlas in Brazil and the Pará State Health Department (SESPA) (19). The data were initially tabulated in Excel spreadsheets after which data mining techniques were used (20, 21). All data sources are publicly available (Supplementary Table S4).

### Statistical analysis

#### Epidemiological rates and stratification

The rates were calculated for the municipalities, as well as for strata within the municipalities, obtained through the classification of patients and inhabitants according to their area of residence (rural and urban), sex, and age group. For mortality rate, we calculated deaths per thousand inhabitants and for hospitalization and fatality, deaths per inhabitant. For descriptive analyses, the age classification used in the demographic census dataset (11 categories) was followed. For data analysis, to obtain sufficient numbers of hospitalization cases per strata for each municipality, we used four age groups. The simplified stratification by age was: (1) From birth to 20 years; (2) From 20 to 40 years; (3) From 40 to 60 years; (4) Over 60 years.

The epidemiological rates were adjusted to control for differences among municipalities in the main effects that could overcome the effects of socioeconomic variables. To study the effects of socioeconomic variables, as well as geospatial patterns, in determining the epidemiological rates, a two-step procedure was applied, to control for effects which influence the determination of these rates, such as age (for example, municipalities with a higher proportion of older people in the population may have higher rates, even if their rates are lower, within each age class).

In the first step, logistic regression analyses were applied to evaluate the magnitude of the effects of the variables, area of residence (urban or rural), sex, age class, and interaction terms, over the variation among municipalities, for each epidemiological index. These analyses used the “*glm*” function, from “stats” package, in R program, with the numerators of the epidemiological rates as the dependent variable, denominators of the epidemiological rates as the weights, and “family” option as “binomial(logit).” The evaluation of the magnitude of each significative effect was carried out by comparing the odds ratios assigned to it (Table 1). This evaluation enabled us to neglect the sex ratio variation among municipalities.

**Table 1:** Logistic regression analysis with effects of sex, age group, and area variables on mortality, hospitalization, and fatality rates due to COVID-19 in the State of Pará, 2020–2021.

Effect	Mortality			Hospitalization			Fatality		
	Odds ratio	Z	*p*	Odds ratio	Z	*p*	Odds ratio	Z	*p*
Intercept	0	−46.53	<2e-16	0	112.53	<2e-16	0.1	−9.92	<2e-16
Urban Zone (U.Z)	1.27	0.92	0.3574	1.83	7.61	3e-14	0.69	−1.35	0.1785
Male Sex (M.S.)	1.17	0.52	0.6024	1.04	0.39	0.6999	1.12	0.37	0.7083
Age range: 20 to 39 (AR.2)	3.54	5.06	4e-07	2.91	13.02	<2e-16	1.21	0.74	0.4600
Age range: 40 to 59 (AR.3)	18.54	12.73	<2e-16	7.98	26.59	<2e-16	2.32	3.48	0.0005
Age range: 60 + (AR.4)	130.64	21.94	<2e-16	29.6	44.95	<2e-16	4.41	6.33	2e-10
U.Z: S.M	1.13	0.35	0.7234	1.03	0.29	0.7750	1.1	0.25	0.8015
U.Z: AR.2	2.03	2.42	0.0155	1.51	4.50	7e-06	1.34	0.96	0.3394
U.Z: AR.3	2.23	2.95	0.0032	1.55	4.96	7e-07	1.44	1.28	0.2003
U.Z: AR.4	2.34	3.22	0.0013	1.45	4.34	1e-05	1.62	1.74	0.0825
M.S: AR.2	0.86	−0.46	0.6488	0.84	−1.50	0.1336	1.02	0.05	0.9600
M.S: AR.3	1.29	0.83	0.4060	1.3	2.44	0.0146	0.99	−0.02	0.9863
M.S: AR.4	1.4	1.14	0.2540	1.42	3.43	0.0006	0.99	−0.04	0.9660
U.Z: S.M: AR.2	1.22	0.51	0.6126	1.37	2.43	0.0152	0.89	−0.27	0.7852 0.8246
U.Z: S.M: AR.3	1	−0.01	0.9935	1.09	0.68	0.4975	0.92	−0.22	
U.Z: S.M: AR.4	0.76	−0.77	0.4427	0.88	−1.11	0.2658	0.87	−0.38	0.7041

Source: Survey Data, 2023. Gray color shade denotes the significant effect on the variables studied.

In the second step, for each municipality, the values of the epidemiological rates for eight strata were calculated, determined by combinations of the two zones (urban and rural) and the four age ranges. The averages of those eight values were applied as the adjusted epidemiological rates. Subsequent analyses were performed with both crude and adjusted epidemiological rates.

#### Multiple regression analysis

For these analyses, variables were previously treated to remove outliers. Secondly, approximation of normality was obtained by application of the Box-cox transformation. Selection of independent variables to be included in the multiple regression analyses was performed, to obtain a set of variables that included different socioeconomic aspects, avoiding the highly correlated variables (22). This selection was based on the inspection of a correlation matrix (Supplementary materials), including epidemiological rates and demographic and socioeconomic variables (population, inhabitants by sex, age, household, Gini coefficient, GDP per capita, HDI, and MHDI), applying the Spearman correlation method, with permutational significance tests. Multivariate analyses were performed by applying the “glm” function, from basic package, in R Program (23).

#### Geo-statistical methods

The geographical analyses of the crude and adjusted rates were carried out at municipal scales, culminating in the formation of clusters of the Amazonian territorial dynamics of COVID-19. To perform the spatial autocorrelation, we used the univariate local Moran’s I index, identifying significant areas of high and low spatial dependence (24). The local spatial association index (LISA) by Queen-type contiguity neighborhood matrix was used to define first-order neighbors, presenting hypotheses of positive or negative autocorrelations, with a spatial statistical significance Lisa *p*-value <0.05, and analyses performed in the GEODA 1.20 software (25). Autocorrelation patterns were classified as High-High, Low-High, High-Low, and Low-Low, according to the Moran diagram. For all analyses, statistical significance was considered at *p*-values <0.05.

## Results

### Exploratory analysis of the variation in crude mortality, hospitalization, and fatality rates of COVID-19 among municipalities in the state of Pará

**Figure 1:**
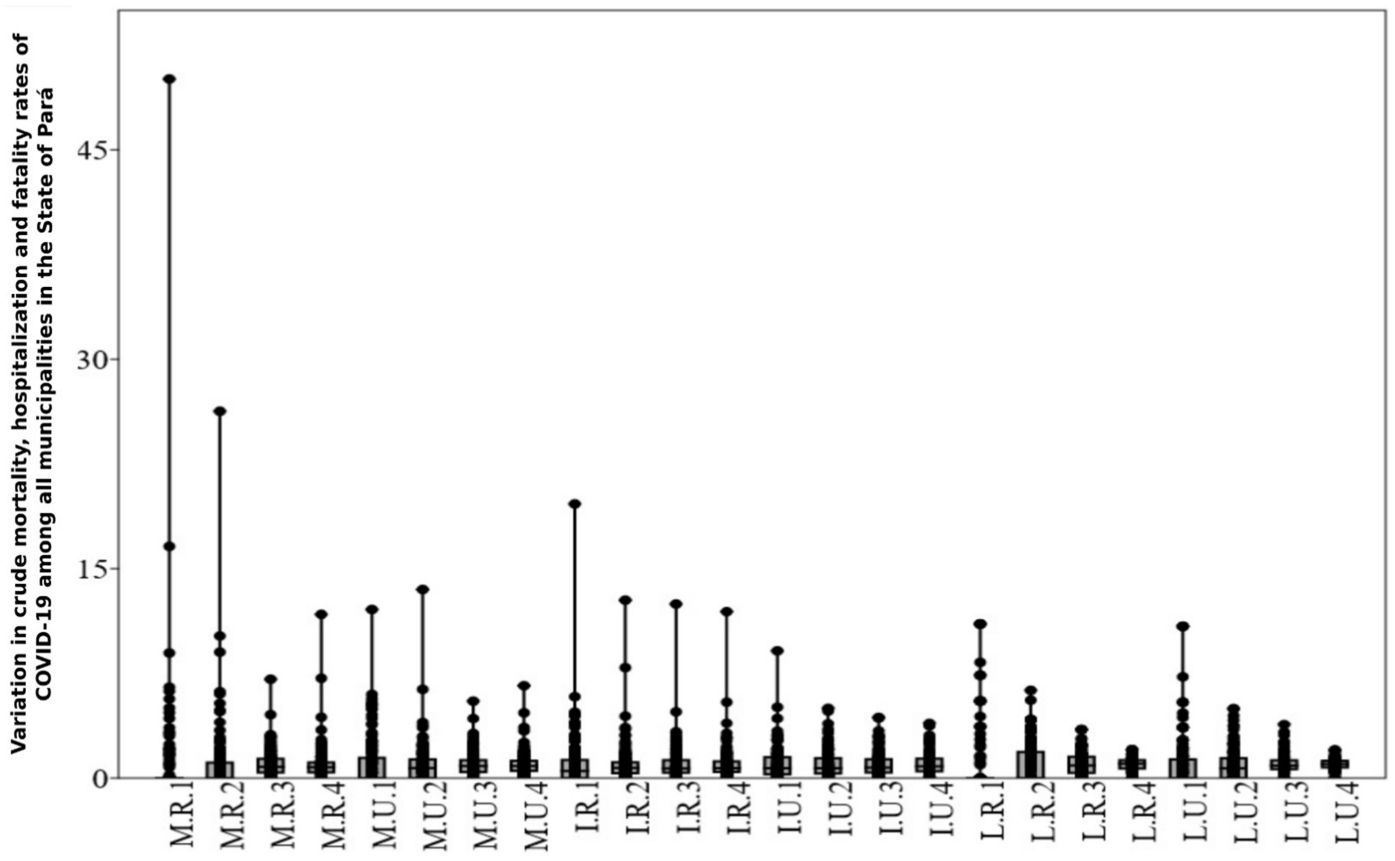
Variation in crude mortality, hospitalization, and fatality rates due to COVID-19 among municipalities in the State of Pará. Source: Survey Data, 2023. M.R.1, rural area mortality, age group 1; M.R.2, rural area mortality, age group 2; M.R.3, rural area mortality, age group 3; M.R.4, rural area mortality, age group 4. M.U.1, urban area mortality, age group 1; M.U.2, urban area mortality, age group 2; M.U.3, urban area mortality, age group 3; M.U.4, urban area mortality, age group 4. I.R.1, rural area hospitalizations, age group 1; I.R.2, rural area hospitalizations, age group 2; I.R.3, rural area hospitalizations, age group 3; I.R.4, rural area hospitalizations, age group 4; I.U.1, urban area hospitalizations, age group 1; I.U.2, urban area hospitalizations, age group 2; I.U.3, urban area hospitalizations, age group 3; I.U.4, urban area hospitalizations, age group 4; L.R.1, rural area fatality, age group 1; L.R.2, rural area fatality, age group 2; L.R.3, rural area fatality, age group 3; L.R.4, rural area fatality, age group 4; L.U.1, urban area fatality, age group 1; L.U.2, urban area fatality, age group 2; L.U.3, urban area fatality, age group 3; L.U.4, urban area fatality, age group 4.

The results of this analysis present an overview of gross COVID-19 rates among Amazonian municipalities (Figure 1). Cases are presented in proportions according to sex, age group, geographic area, and deaths. In graph 1 below, the x-axis demonstrates the rates for the municipalities, calculated from the average of all municipalities, and the y-axis demonstrates the mortality (M), hospitalization (H), and fatality (F) rates, separated according to the indication of urban (U) or rural (R) areas, and age groups 1, 2, 3, and 4 (0 to 19 years; 20 to 39 years; 40 to 59 years; over 60 years respectively). It should be noted that each point on the graph corresponds to a municipality.

The variation in the crude mortality rate in rural areas, in age group 1 (M.R.1), was 456.7%, the highest among the rural (R) and urban (U) age groups. The absence of deaths in this age group in 116 municipalities may have influenced this result. Of the 28 municipalities where deaths were recorded, *Pau D’Arco, Jacareacanga*, and *Mocajuba*, showed the greatest variations (2.849, 0.944, and 0.509 per thousand inhabitants respectively). However, in the urban area (U), *Pau D’Arco* and *Jacareacanga* did not record deaths in age group 1. The greatest variation in the crude mortality rate in rural areas in age group 2 (M.R.2) was 265.3%, followed by age group 4, with 124.6% among municipalities. The crude mortality rate in the urban area (M.U) among municipalities followed the pattern of the rural area (R), with the greatest variation being observed in age groups 1 and 2 (177.1 and 143.2% respectively).

The variation in the gross hospitalization rate in rural areas (H.R) was higher for all age groups (119–191.7%), when compared to hospitalizations in urban areas (H.U; 73.3–114.1%).

The variation in the crude fatality rate in rural areas (F.R) was higher in age groups 1 and 2 (246.7 and 140.6% respectively). In urban areas, the fatality rate (F.U) varied between 35.3 and 199.9%, repeating the pattern of age groups 1 and 2 in rural areas (F.R).

The variations in the crude rates (Figure 1) above provided an overview of the dispersion of the data and helped to identify the outlier municipalities (Table 2). Scientific evidence shows that sex, age (26), and population density in urban areas have a large effect on COVID-19 infection rates and severity. Therefore, it is plausible to consider these effects, as well as control for them, in analyses targeting COVID-19 epidemiological rates.

**Table 2:** Upper outlier municipalities for crude and adjusted mortality, hospitalization, and fatality rates for COVID-19 in the State of Pará, 2020–2021.

Municipalities outliers superior (>75%)	Mortality rate	Hospitalization rate	Fatality rate
Crude rates	*Jacareacanga, Santarém, Canaã dos Carajás, Faro*	*Canaã dos Carajás, Faro, Brasil Novo, Rio Maria*	There were no municipalities with a value higher than 75%
Adjusted rates	*Jacareacanga, Juruti, Santarém, Canaã dos Carajás, Faro*	*Jacareacanga, Canaã dos Carajás, Faro, Brasil Novo, Rio Maria*	*Acará* and *Belterra*

Source: Survey Data, 2023.

The following logistic regression analyses (Table 1) allow us to examine the effects of variations in sex, area, and age groups on the epidemiological rates of the municipalities.

The results presented as odds ratios represent the relative risk of an individual in a given category being exposed, compared to an unexposed individual. These values are estimates of relative risk, i.e., the comparison of data between the variables studied, urban versus rural areas, male versus female, and the values for the three age groups listed in the table compared to the age group not included in the table, which is up to 19 years of age.

The results of the logistic regression (Table 1), as well as the coefficients of variation obtained for the three independent variables analyzed (Figure 1), show the differences between the municipalities in terms of age structures and proportions of the populations that are in rural or urban areas, and that have the potential to cause relevant variations in the municipalities’ COVID-19 rates. Although the effect of the areas in this analysis was not very significant, the high coefficient of variation in the distribution of the population between rural and urban areas, a characteristic of the State of Pará, suggests the need to control its effect in subsequent analyses.

### Spatial clusters of COVID-19 in the state of Pará

**Figure 2:**
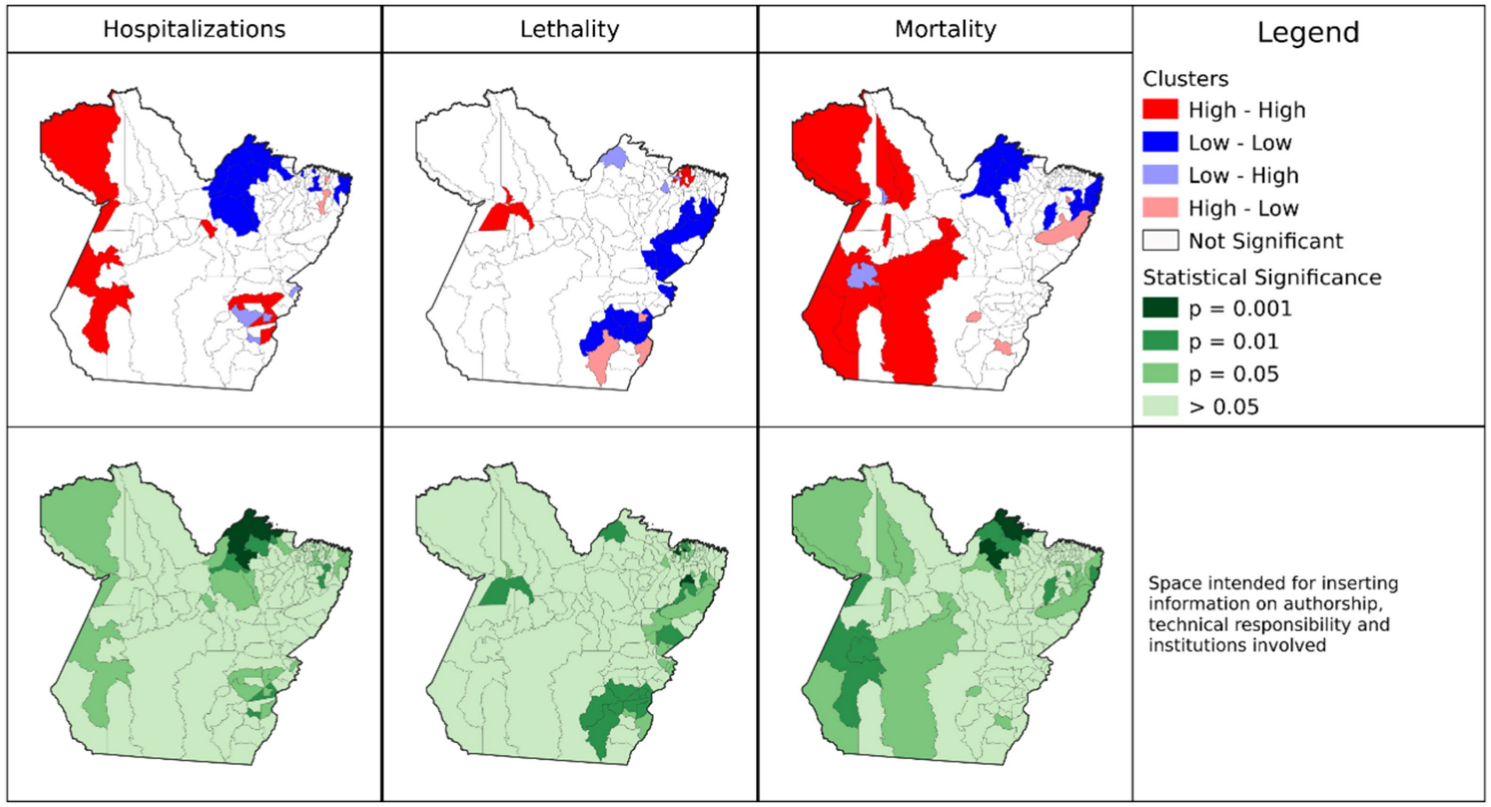
Spatial autocorrelation clusters of crude mortality, hospitalization, and fatality rates of COVID-19 in municipalities of the State of Pará, 2020–2021. Source: Survey Data, 2023.

**Figure 3:**
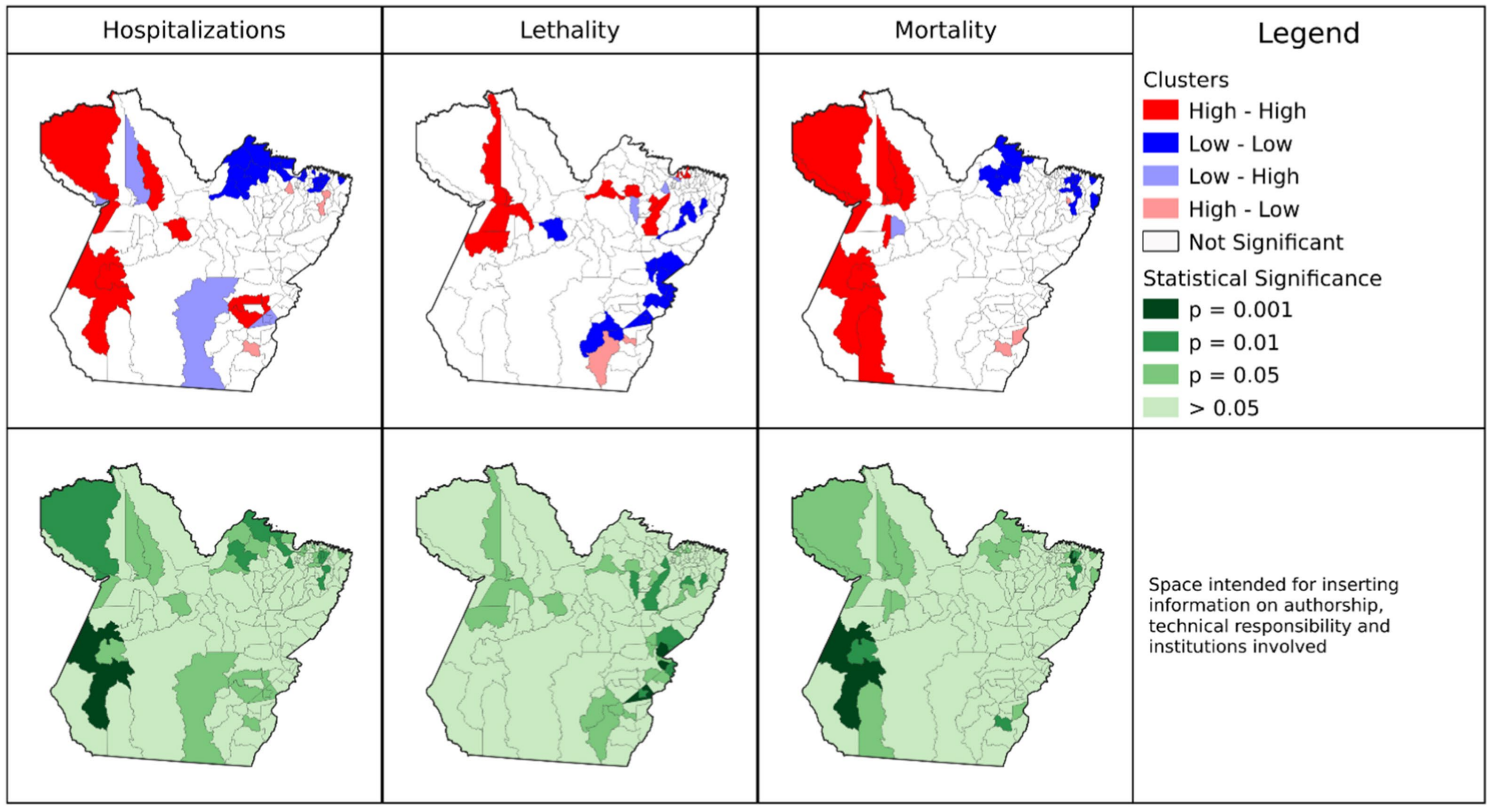
Spatial autocorrelation clusters of adjusted mortality, hospitalization, and fatality rates of COVID-19 in municipalities of the State of Pará, 2020–2021. Source: Survey Data, 2023.

In the analyses based on crude COVID-19 mortality rates, 11 municipalities in the *Tapajós* (*Itaituba*), Baixo Amazonas (*Alenquer, Belterra, Faro, Juriti, Monte Alegre, Oriximiná*, *Terra Santa*), and Xingu (*Altamira, Brasil Novo*) regions formed clusters with a High-High classification for mortality (Figure 2). In the adjusted rates (Figure 3), three municipalities (*Altamira, Brasil Novo*, and *Jacareacanga*) did not present spatial statistical significance and the municipalities of *Curuá, Novo Progresso*, and *Trairão* became part of the cluster.

The results indicate spatial dependence of *Jacareacanga* and *Altamira* with neighboring municipalities, influencing their final classification. We identified discrepancies in values between sampling units, and municipalities whose crude rate data exceeded 75% (outliers; Table 2) and that have spatial dependence. The hypothesis raised is that high gross hospitalization rates in municipalities disproportionate to what is expected in the population may have generated the outliers.

Nine municipalities (*Augusto Correa, Bonito, Capanema, Igarapé-Açu, Nova Timboteua, Ourem, Peixe-Boi, Primavera*, and *Santarém Novo*) located close to *Belém*, the state capital, formed clusters of low adjusted mortality rates (Figure 3). Three municipalities (*Redenção, Floresta do Araguaia*, and *Mãe do Rio*) were considered “transition zones” (High-Low), with high mortality rates and neighbors with low rates, or did not present the same spatial dependence cooperating for negative autocorrelation. The remaining 113 municipalities did not show spatial statistical significance. However, very high and positive values indicate the presence of clusters, and low values demonstrate spatial inequality in the region or transition zones.

The municipalities of *Juruti, Itaituba, Curionópolis, Parauapebas, Oriximiná, Monte Alegre*, *Medicilândia, Trairão*, and *Água Azul do Norte* formed clusters of high adjusted hospitalization rates (High-High). Of these, *Juruti, Itaituba, Oriximiná, Monte Alegre*, and *Trairão* were significant for the mortality rate. In this analysis, four municipalities (*Santo Antônio do Tauá, Vigia, São João da Ponta*, and *Melgaço*) are “transition zones” for one or more rates, as they are areas that contributed to positive autocorrelation, that is, the clusters of these last three municipalities reflect areas with low hospitalization rates and high fatality (Low-High) but are not statistically significant for mortality rates. The municipalities of *Barcarena, Capitão Poço*, and *Redenção* are transition zones with high hospitalization rates and low fatality rates (High-Low). Finally, 104 municipalities presented results that were not significant for the adjusted hospitalization rate.

The municipalities, *Terra Alta, São João da Ponta, Santarém, Curuá, Marapanim, Curralinho*, *Igarapé-Miri, Moju, Aveiro, Vigia, Óbidos*, and *Melgaço*, formed clusters of high adjusted fatality rates (High-High), however, *Curuá* had both high fatality and mortality rates, and was not significant for hospitalization. The municipalities of *Cumaru do Norte* and *Pau D’Arco* are considered transition zones for adjusted COVID-19 case fatality rates (High-Low). In total, 108 municipalities presented non-significant results for fatality rate (Lisa *p* > 0.05).

For all crude rates, the municipality of *Afuá* presented an intersection with a significant result for the three rates studied (Supplementary Figure S2). However, this area presented low values for hospitalization and mortality (Low-Low), and for fatality (Low-High). These results have positive values, however, the municipality presented a negative average, with the distinct high values among its neighbors reflecting in the fatality result, indicating that this is a transition point between local spatial patterns. However, for the adjusted rates, the classification of *Afuá* changes and it becomes insignificant (Supplementary Figure S3).

There were no records of municipalities with simultaneous intersection of high values for the three rates studied (Supplementary materials), indicating that the result in the crude rates can be attributed to the effects of age group, area of residence, or relationship with neighboring municipalities. We highlight the need for more detailed study of the municipalities that were identified as transition zones in the geospatial analyses.

### Socioeconomic predictors of COVID-19 outcomes

Tables 3, 4 present the interaction of epidemiological rates with demographic and socioeconomic variables. The results of Spearman’s correlation coefficients (Table 3) are similar for mortality and hospitalization, whether for crude or adjusted rates. However, the fatality rate interacts differently from the other two rates. In the three adjusted rates, only GDP per capita showed a strong significant correlation with the crude (*ρ* = 0.53) or adjusted (ρ = 0.44) hospitalization rate. The other variables did not present significant correlations.

**Table 3:** Spearman correlation coefficients between demographic and socioeconomic variables and crude and adjusted rates of hospitalization, fatality and mortality from COVID-19 in the State of Pará.

Demographic and socioeconomic variables	Crude rates			Adjusted rates		
	Mort.	Int.	Let.	Mort.	Int.	Let.
AREAMED	−0.04	0.20*	−0.40*	0.01	0.20*	−0.32*
Population density	0.06	−0.04	0.12	−0.06	−0.06	0.04
Population	0.16	0.18*	0.14	−0.17*	−0.03*	−0.17*
GDP per capita	0.48*	0.53*	−0.19*	0.36*	0.44*	−0.07
Gini coefficient	−0.27*	−0.34*	0.21*	−0.29*	−0.38*	0.21*
MHDI 2010	0.44*	0.39*	−0.08	0.14	0.19*	−0.08
MHDI Educa2010	0.11	0.12	−0.02	0.40*	0.30*	0.01
MHDI Longev2010	0.22*	0.24*	−0.03	0.32*	0.29*	−0.03
MHDI Income2010	0.40*	0.42*	−0.18*	0.15	0.24*	−0.15
Adequate sanitation	0.00	0.00	0.04	0.17*	0.06	0.07
%Inhabitants/household inadequate	0.01	−0.12	0.18*	−0.21*	−0.26*	0.12
Residents per household	0.12	−0.07	0.30*	−0.14	−0.29*	0.19*

*Significant results (Αα = 0.05). Positive correlations are shown in blue and negative correlations are shown in orange. MHDI 2010, Municipal Human Development Index, year 2010; MHDI Educa2010, education component; MHDI Longev2010, longevity component; MHDI Income2010, income component; %Inhabitants/homes inadequate, percentage of inhabitants with inadequate housing. Source: Survey Data, 2023.

**Table 4:** Multiple regression of adjusted mortality, hospitalization, and fatality rates of COVID-19 with demographic and socioeconomic variables in municipalities in the State of Pará.

Effects	Mortality		Hospitalizations		Fatality	
	*β*	Pr(>|t|)	*β*	Pr(>|t|)	*β*	Pr(>|t|)
Intercepts	−0.04	0.716	−0.04	0.658	−0.00	1.000
POP	−0.05	0.655	0.08	0.523	0.07	0.455
GDP	0.32*	0.005*	0.35*	0.002*	0.03	0.743
Residents	0.20	0.140	−0.00	0.997	0.36*	0.002*
Gini	−0.19	0.054	−0.32*	0.004*	0.17*	0.040*
HDI	0.15	0.344	−0.08	0.628	0.16	0.207
POP:GDP	−0.11	0.440	−0.24	0.093	–	–
POP:Residents	0.07	0.585	0.04	0.775	–	–
POP:Gini	−0.04	0.729	−0.01	0.920	–	–
POP:HDI	−0.13	0.347	0.12	0.411	–	–
GDP:Residents	0.23	0.090	0.20	0.135	–	–
GDP:Gini	−0.12	0.277	−0.20	0.096	–	–
GDP:HDI	0.40*	0.009*	0.17	0.262	–	–
Residents:Gini	0.07	0.593	0.04	0.809	–	–
Residents:HDI	−0.02	0.842	−0.14	0.194	–	–
Gini:HDI	0.26	0.070	0.04	0.772	–	–
POP:GDP:Residents	−0.23	0.030	−0.22	0.065	–	–
POP:GDP:Gini	−0.34*	0.008*	−0.02	0.895	–	–
POP:GDP:HDI	0.02	0.921	0.16	0.352	–	–
POP:Residents:Gini	−0.30*	0.018*	−0.20	0.195	–	–
POP:Residents:HDI	0.15	0.378	0.53*	0.003*	–	–
POP:Gini:HDI	0.05	0.688	−0.05	0.779	–	–
GDP:Residents:Gini	0.04	0.762	−0.04	0.814	–	–
GDP:Residents:HDI	−0.08	0.304	−0.19*	0.010*	–	–
GDP:Gini:HDI	−0.05	0.656	0.13	0.363	–	–
Residents:Gini:HDI	−0.15	0.176	0.04	0.754	–	–
POP:GDP:Residents:Gini			0.04	0.756	–	–
POP:GDP:Residents:HDI			0.07	0.478	–	–
POP:GDP:Gini:HDI			−0.08	0.690	–	–
POP:Residents:Gini:HDI			−0.39*	0.049*	–	–
GDP:Residents:Gini:HDI			−0.05	0.580	–	–

*Significant results (Αα = 0.05). Positive correlations are shown in blue and negative correlations are shown in orange. POP, population; GDP, Gross Domestic Product; Residents, number of residents per household; HDI, Human Development Index; GINI, GINI index. Source: Survey Data, 2023.

In the multiple linear model, all variables demonstrated a significant association with mortality and hospitalization rates, either in the isolated effect or within interaction terms (Αα = 0.05). However, only GDP had a significative isolate association with mortality and hospitalization. Surprisingly those associations were positive, meaning that mortality and hospitalization rates were higher in municipalities with higher GDP. On the other hand, only residents and Gini index had significative isolate associations with fatality, being those associations also positive. The only negative isolate effect was detected for the association between Gini index and hospitalization rates (Table 4).

**Figure 4:**
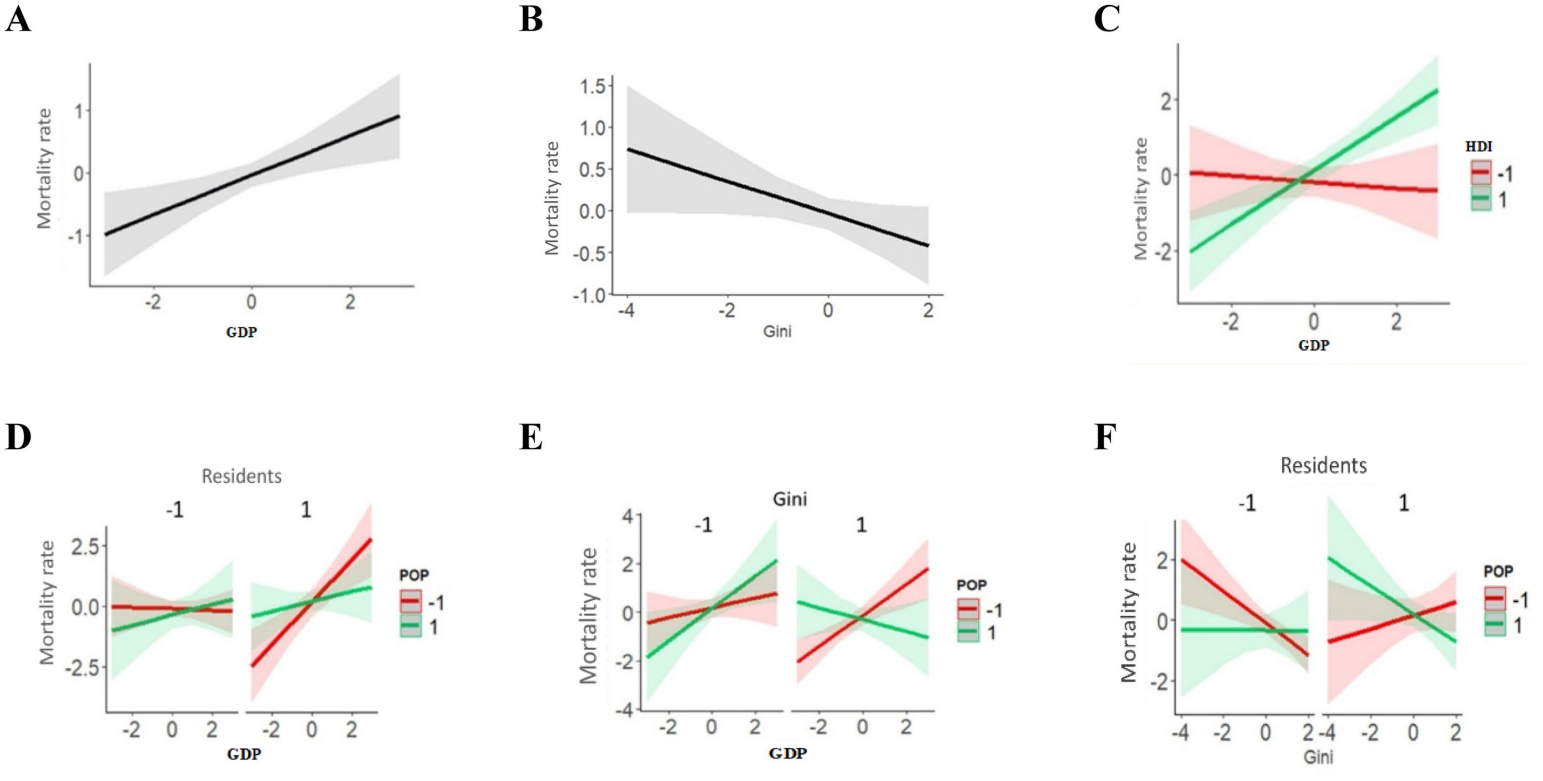
Multiple logistic regression analysis and socioeconomic variables on adjusted COVID-19 mortality rates in municipalities of the State of Pará 2020–2021. **(A)** Isolated effect of GDP on Mortality rates. **(B)** Isolated effect of Gini Index on Mortality rates. **(C)** Interaction effect between GDP and HDI on Mortality rates. **(D)** Interaction effect between Residents, GDP and POP on Mortality rates. **(E)** Interaction effect between Gini, GDP and POP on Mortality rates. **(F)** Interaction effect between Residents, Gini and POP on Mortality rates.

The results of the multiple regression, including the interaction effects, can be inspected on Figure 4. The effect of each variable may have an inverted sign (+ or –), depending on the characteristics of the municipalities in relation to the other variables tested. Despite this, for the variables GDP and Gini coefficient it was possible to identify a predominant direction for associations with mortality rates.

The positive association between GDP and mortality rates indicates that municipalities with a higher GDP had higher rates. However, the Gini coefficient showed a marginally significant negative association (*p* = 0.054), meaning that municipalities in Pará with greater income equality are associated with lower mortality rates from COVID-19.

The GDP and HDI variables (Figure 4C) interact due to the positive association of GDP with mortality only in municipalities with the high HDI. The interaction between Residents, Population, and GDP (Figure 4D) can be summarized as the positive association of GDP with hospitalization being higher in municipalities with a smaller population and more residents per household.

The interaction between GDP, the Gini coefficient, and POP (Figure 4E) consists of the inversion of the association between GDP and mortality rate in municipalities with higher values for the Gini coefficient and population size. The interaction between the Gini coefficient, Residents, and POP (Figure 4F) results from the inversion of the association between the Gini coefficient and mortality among municipalities with more residents per household and a smaller population.

**Figure 5:**
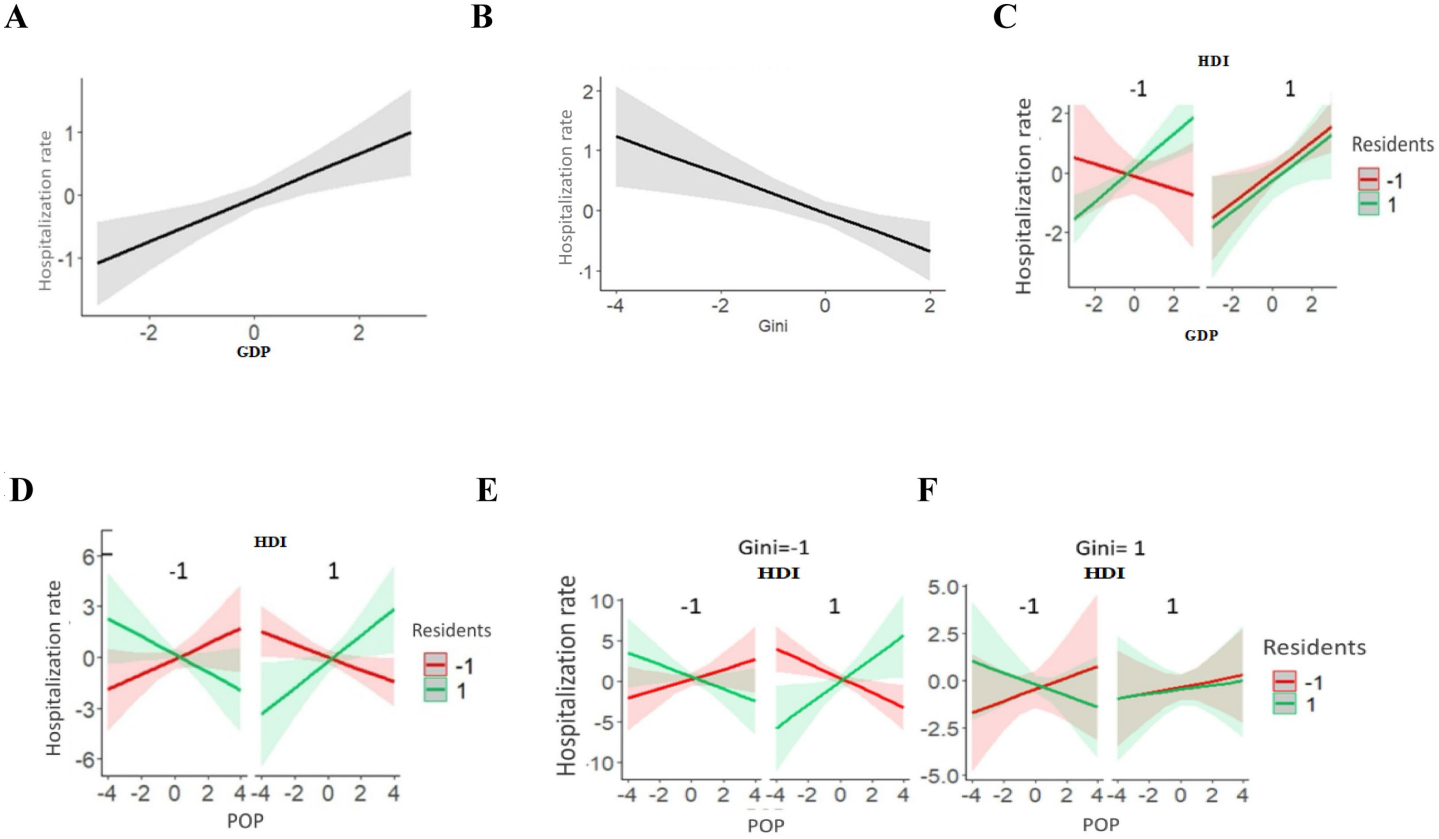
Multiple logistic regression analysis and socioeconomic variables HDI, POP, residents, GINI on the COVID-19 adjusted hospitalization rate in municipalities of the State of Pará 2020-2021. **(A)** Isolated effect of GDP on Hospitalization rates. **(B)** Isolated effect of Gini Index on Hospitalization rates. **(C)** Interaction effect between HDI, GDP and Residents on Hospitalization rates. **(D)** Interaction effect between HDI, POP and Residents on Hospitalization rates. **(E)** Interaction effect between HDI, POP, Residents and Gini on Hospitalization rates, for low values of Gini. **(F)** Interaction effect between HDI, POP, Residents and Gini on Hospitalization rates, for high values of Gini.

The adjusted hospitalization rate, GDP, and Gini coefficient (Figures 5A,B) showed significant isolated effects. A higher GDP was associated with a higher hospitalization rate and the Gini coefficient showed the opposite pattern (lower income inequality and lower hospitalization rate).

In line with the results of the mortality rate, significant interaction effects were detected for the hospitalization rate in all variables tested. The interaction of GDP, Residents, and HDI (Figure 5C) results from the positive association of GDP with the hospitalization rate and is reversed in municipalities with low HDI and fewer residents per household.

The interaction between POP, Residents, and HDI (Figure 5D) indicates that a change from low to high values in any of these three variables reverses the association of the other two with hospitalization rates. The interaction between Population, Residents, HDI, and the Gini coefficient (Figure 5E) consists of the addition of the Gini coefficient to the interaction mentioned and two changes are observed in municipalities with higher Gini coefficients: (1) Population size loses effect (larger confidence intervals); (2) Among municipalities with a higher HDI, the number of residents per household loses effect.

**Figure 6:**
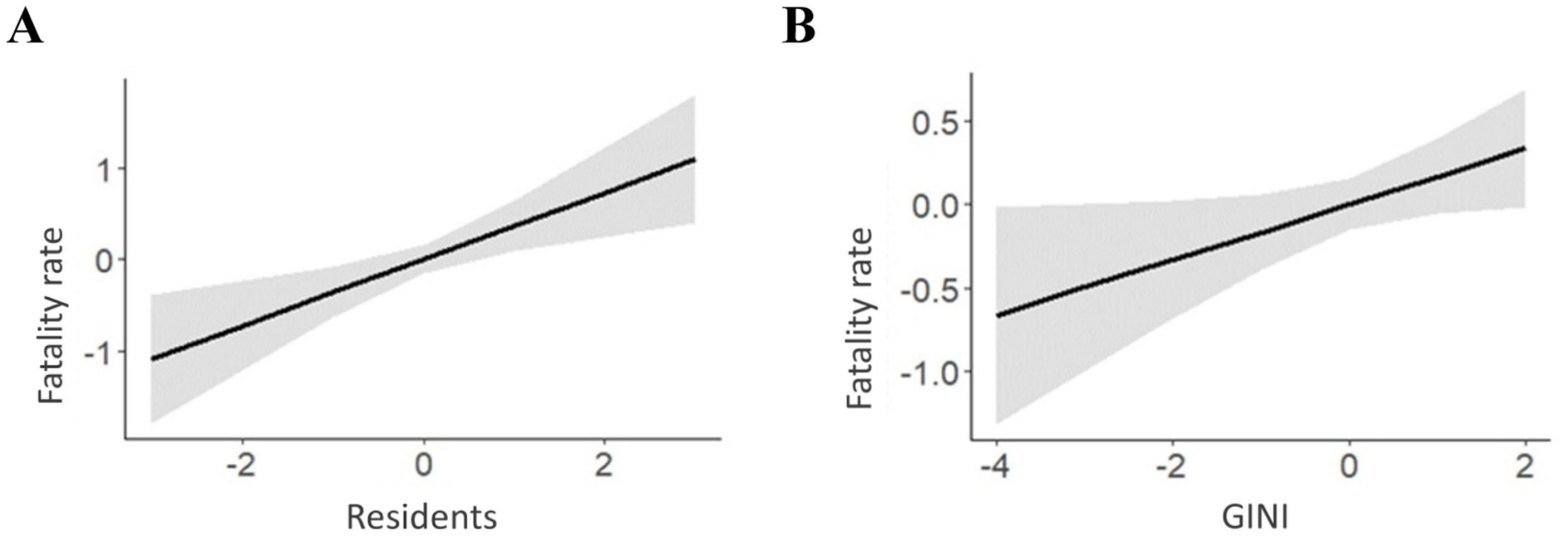
Multiple logistic regression analysis and socioeconomic variables residents, GINI on the adjusted fatality rate of COVID-19 in municipalities in the State of Pará 2020-2021. **(A)** Isolated effect of Residents on Fatality rates. **(B)** Isolated effect of Gini Index on Fatality rates.

For the fatality rate, Residents and the Gini coefficient (Figure 6) present significant isolated effects on hospitalization rates, both positively associated with the fatality rate, i.e., municipalities with a greater number of residents per household or a higher Gini coefficient, had higher fatality rates. It is worth noting that the number of residents per household is strongly associated with the fatality rate, as it presents a higher *β* value, as previously explained (Table 4).

## Discussion

Environmental changes, especially climate changes, raise concerns about meteorological factors in the transmission of COVID-19, since climate systems are stochastic, dynamic, and complex, requiring regional knowledge, preparation of local communities, and improved response capacity. Environmental tensions underscore the urgency of monitoring Amazonian issues of territory, population, services, and regional public health policies, considering the influence on cyclical respiratory infections such as COVID-19 (27–30).

**Figure 7:**
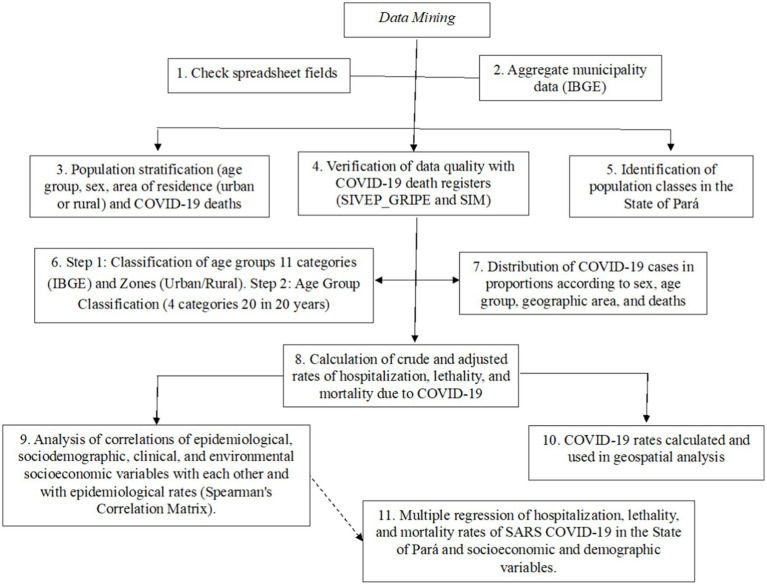
Study methodology flowchart. Source: Authors, 2023.

We detected discrepancies in the variation in gross mortality, hospitalization, and fatality rates among Amazonian municipalities, considering the age group and area of residence (Figure 7). The patterns found in this first analysis reveal large differences between municipalities and age groups, especially among children and adolescents, newborns, and early childhood. However, there was an absence of deaths in certain municipalities and in specific age groups. This result may have generated outliers (Table 2), as well as being associated with issues related to the age pyramid of the population in that municipality or underreporting that affected the quality of records during the pandemic.

Global underreporting of deaths in several countries with the gross risk of fatality was attributed to differences found in the availability and capacity of testing, containment, infrastructure, and general medical care (31). Significant differences in SARI deaths were also found in the cities of Manaus, Belém, Fortaleza, Recife, São Paulo, and Rio de Janeiro, with variations between 493% and 5,820%, values considered very high. The authors attributed the high value to reporting errors, estimating an average underreporting of 40.68% (range 25.9 to 62.7%) of deaths related to COVID-19 (32).

Scientific evidence shows that sex, age (26), and population density in urban areas have a large effect on COVID-19 infection rates and severity. Therefore, it is plausible to consider these effects, as well as control for them, in analyses targeting COVID-19 epidemiological rates. The use of age-standardized mortality rates was recommended by researchers, with the aim of eliminating interpretative biases, especially in the northern region of Brazil (33), corroborating our results (Table 1).

In the current study, the rates of COVID-19 geographically distributed among municipalities in the State of Pará were shown to be significant in those close to the border with the States of Amazonas and Amapá. Of particular note is the municipality of *Itaituba*, which has human mobility by river and road, and is dependent on highly complex health services in the hub city of *Santarém.*

Among the municipalities analyzed, the municipality of *Santarém* had a high fatality rate, despite having the best healthcare infrastructure in the *Baixo Amazonas* and *Tapajós* region. However, the results for adjusted mortality and hospitalization rates were not significant. The high fatality rate in this municipality may have been caused by the “migration of patients from neighboring municipalities,” without adequate conditions for medium and high complexity hospital support, overcrowding of ICU beds, and/or the delay in medical assistance, impacting hospital deaths, especially in patients with associated comorbidities or a serious condition (34, 35).

Another relevant factor was the supply of equipment, as the poor distribution of ventilators and ICU beds in the state culminated in a shortage of resources in certain areas, according to the results presented in the study by Rezende and cols (36).

In the crude rates, the municipalities of *Jacareacanga, Canaã dos Carajás*, and *Acará* (outliers) presented high mortality and hospitalization rates. It is possible that the high hospitalization rates may have generated outliers. However, in the municipality of *Afuá*, located on the island of *Marajó*, which has a scenario of extreme poverty and child sexual exploitation, low fatality rates were observed, which may be related to the characteristics of the regional geography in this area.

The precariousness of records and issues related to the management of the notification or testing service may attenuate results regarding COVID-19 rates in the territory and it is worth noting that these effects were not included in the current study. However, there is a need to investigate in detail the municipalities that are transition zones in geospatial analyses.

Municipalities in the Amazon use roads and/or rivers as a means of transportation, as they are located on the banks of Amazonian rivers and have difficult access roads. For example, *Jacareacanga*, which is located on the banks of the *Tapajós* River, is a tourist region with mining as its main economic activity, and has more than 100 indigenous villages, with the largest proportion of this population in the State of Pará (17). This region has the lowest socioeconomic indices in Brazil, according to Social Progress Imperative Amazônia 2021 (IPS = 46.83), displaying a scenario of socio-environmental vulnerability (37).

In May 2020, the states of Amazonas and Pará were the most affected in the spatial distribution of registered cases of COVID-19, and the capital of Pará had the highest incidence (1,816.4/1,000,000 inhabitants) and mortality (240/1,000,000 inhabitants), with a mortality rate of 9.9% (38). At the height of the COVID-19 pandemic, health services in the State of Amazonas were overwhelmed, a fact publicized in the press due to the circulation of the P1 variant (39).

Some researchers warned that river routes were driving the “spread of contagion” within the state of Amazonas and other regions, and they studied the mobility and evolution of COVID-19, suggesting that many trips departing from *Manaus* could increase the likelihood of the disease spreading from the epicenter to other regions, with infected people entering the destination city, and accelerating the progression of the pandemic in that population. The authors cite as examples changes in the incidence rates of municipalities that are 985 km away (*Itamarati*) within the state itself (40).

We observed in this study that border municipalities may have high epidemiological rates (*Jacareacanga*) or low rates (*Afuá*) depending on their geographic location, means of transportation, and feasibility of access to health services. A study using data from 48 Spanish provinces, from August 2020 to March 2021, found a high pattern of correlations between the growth rate of COVID-19 and mobility and meteorological data, based on three or four time series of data (R^2^ = 0.65). The authors found a greater influence of mobility on the spread of COVID-19 (27).

The pandemic has exposed the complexity of the spatial delimitation of contagion units, due to the scalar paradox of the disease (global contamination and local response), and makes it imperative to map the possible circuits for decision-making, especially in border areas (41).

Researchers tracked community incidence and mortality records for COVID-19 in the State of Pará, after implementing social distancing through quarantine and lockdown. The authors observed that it took 49 days for 81% of the state’s 144 municipalities, spread over an area of approximately 1,248,000 km^2^, to register cases of COVID-19. Indicating that short periods of lockdown may have promoted the spread of the virus between municipalities on the outskirts of the capital and regions in the interior (42).

Data demonstrate that states with large territorial areas contributed to delays in notifications and an increase in cases. In addition, environmental factors are determining factors in the infection, distribution, and transmission of the virus, with different behavior depending on the region and culture. Furthermore, economic aspects associated with socio-spatial vulnerability can make territories more susceptible to potential moderate or strong risks depending on local geographic conditions and proximity between territories (38).

The pandemic has highlighted that social and territorial inequalities influence geographic dynamics. It is estimated that the global inequality caused by COVID-19 has affected the Gini coefficient, increasing it between countries by up to 0.3%. This effect was caused by the decline in global consumption of non-essential sectors in richer countries, which impacted GDP and employment in low-income countries. For every 1% decline in demand for non-essential goods, an increase in global inequality is expected, and less diversified economies are more vulnerable to these global shocks (43).

The North region contains municipalities with high levels of social inequality and great socio-ecological vulnerability, that were aggravated by the COVID-19 pandemic (13). A study analyzed Amazonian municipalities with up to 50,000 inhabitants, considering social, environmental, and economic aspects, to determine the exposure of the population and environment, as well as vulnerability in the event of extreme events. The authors found that the factors that most increased the degree of vulnerability of municipalities were precarious urban mobility, lack of medical services, difficulty in accessing the internet and means of communication; the services most used by the population in emergencies. Of the 265 municipalities considered highly vulnerable, Pará contains 73 and concentrates the 11 with the highest index in the survey (44).

A key point for managing a health crisis is a data generation policy that shows the different local realities, adding information for decision-making during the emergency. The lack of regionalized studies on the evolution of the COVID-19 pandemic has made it difficult to respond quickly and effectively, with integrated and personalized actions for the different epidemiological contexts.

The initial spread of infections and deaths from COVID-19 in Brazil was more affected by patterns of socioeconomic vulnerability than by the age structure of the population and the prevalence of morbidity from existing chronic diseases; mortality rates increased rapidly, especially in the North and Northeast regions (45).

Several studies associate higher rates of infection and deaths from COVID-19 with social, economic, and demographic factors, with populations living in more vulnerable environments being at a greater risk when compared to groups with greater economic power, that is, disparities in demographic and economic conditions are important for SARS-CoV-2 virus infection rates (46, 47). The economic recession caused by the pandemic has affected the low-income population, with job losses and reduced demand for services, and even the new demands arising with remote work have not affected this group, especially in the Amazon where connectivity and digital inclusion are still a huge challenge.

In the current study, the socioeconomic variables GDP and the Gini index showed significant isolated effects on the adjusted hospitalization rate in the municipalities studied, an effect also identified for the mortality rate. Municipalities with a higher GDP were associated with higher hospitalization rates and those with lower income inequality were associated with lower hospitalization rates.

The results obtained from multiple regression highlight the complexity of the relationships between socioeconomic variables and epidemiological rates; for analyses we remove the effect of age in the population studied. We identified a positive association between GDP and mortality and hospitalization rate, which indicates a trend towards higher rates in municipalities with higher GDP. In the results of the analysis of the association between GDP and mortality, it can be observed that: (1) This main trend is present only in municipalities with higher HDI values (Figure 4C), based on results on the interaction between GDP and HDI, and may be related to particularities of the Amazon region, such as the way in which the population travels along river routes in small and medium-sized boats, favoring the spread of the SARS-CoV-2 virus among vulnerable riverside populations (41); (2) The positive association between mortality and GDP is clearer in municipalities with small populations, but with a high number of people per domicile. A regional characteristic is that high average incomes result from certain economic sources, such as large mining companies, since mining accounts for a third of the Pará State economy (48).

Similar results were found by Xavier et al. (49), for whom this interaction between GDP and the highest COVID-19 mortality rate involves political, behavioral, health infrastructure, and income inequality issues. However, the “hypotheses” raised in the current study suggest that the use of less or more restrictive criteria in Amazonian municipalities for hospitalizing a patient, the severity of their condition, the quality of care, adequate conditions for managing and treating the disease, travel time to the unit, delay in hospital care, and availability of beds, in addition to issues related to records in the system, are some of the regional factors that can affect the results of COVID-19 mortality and fatality rates, and are therefore, multifactorial.

Researchers have verified the community incidence and mortality records for COVID-19 in the state of Pará, after the implementation of social distancing and lockdown. The results showed that 81% of the 144 municipalities took 49 days to register cases of COVID-19. The authors affirm that states with large territorial areas contributed to delays in notifications and an increase in cases, in addition to which, environmental factors are determinants in the infection, distribution, and transmission of viruses, and have different behavior according to the region and culture (50).

The “transition zones” highlighted in the current study show the pandemic dynamics in the Amazon territory, indicating areas that could be “sentinels,” either due to the results of high or low epidemiological rates of COVID-19, as both results are indicators of the management of the health crisis in the municipalities and can signal the most appropriate level of response in future infectious disease emergencies. It is worth noting that the results of this study present the COVID-19 scenario in a time frame of the ongoing pandemic and the information generated evaluates how the Pará territory was affected by COVID-19, expands the analytical capacity of the event, and contributes to future collaborative surveillance between states.

### Limitations

The current study has limitations inherent to the use of secondary data, whose values may contain underestimations. The analyses carried out in this study do not consider the effects of vaccination, and the population estimates used refer to the 2010 Demographic Census (IBGE). The results obtained can support the implementation and planning of health policies, partnerships in collaborative surveillance, and new scientific research.

## Conclusion

The variations in crude mortality, hospitalization, and fatality rates showed different patterns among Amazonian municipalities and age groups, mainly among children and adolescents, newborns and early childhood, and areas of residence. Although the effect of the area of residence was not significant in the analysis, the high coefficient of variation in the distribution of the population between rural and urban areas, a characteristic of the State of Pará, suggests the need to for control its effect.

Spatial analysis of COVID-19 in the State of Pará identified municipalities that are transition zones between low and high mortality, hospitalization, and fatality rates. Clusters of the most affected municipalities showed that there was no overlap of the three epidemiological rates of COVID-19 in the same municipality. However, the socioeconomic variables GDP, the Gini index, and number of residents per household were significant, impacting the behavior of the rates.

Of note are the transition zones made up of the municipalities *Floresta do Araguaia, Mãe do Rio*, and *Redenção* for mortality, *Barcarena, Capitão Poço*, and *Redenção* for hospitalization, and *Cumaru do Norte* and *Pau D’Arco* for fatality, aiming to understand the health dynamics in each territory. It is worth noting that all identified clusters are relevant for future more detailed analyses. Finally, we recommend the adoption of strategies customized to local conditions according to the different territories, since the areas most affected by mortality and fatality are located in regions of greater socio-environmental vulnerability and close to the border zone with the State of Amazonas.

Finally, we recommend further studies in the Tapajós and Baixo Amazonas integration region, considering its territorial and social configuration, as well as the adoption of strategies tailored to local conditions according to the different territories, since the most affected areas were located in regions of greater socio-environmental vulnerability and close to the border zone with the State of Amazonas (51).

## Data Availability

The data and materials that support the findings of this study are available from the corresponding author upon reasonable request.
