# Supplementary material for: Spatial and socioeconomic patterns of COVID-19 in transition zones between municipalities in eastern Amazonia
Source: Front Public Health. 2025 Mar 13;13:1526642. doi: 10.3389/fpubh.2025.1526642 (PMC11966966; doi:10.3389/fpubh.2025.1526642)
Supplement: Supplementary file 2 [file Supplementary_file_1.pdf]

Supplementary Material

Supplement to Vasconcelos et al: Spatial and socioeconomic patterns of COVID-19 in transition zones between municipalities in Eastern Amazonia, 2020-2021.

Contents

Figure S1: Map of the geographic location of the Integration Regions of the State of Pará (R.I)

Table S1: Identification of municipalities according to population size classes by number of inhabitants in the State of Pará.

Table S2**:** Spearman correlation matrix of COVID-19 mortality, hospitalization and fatality rates and socioeconomic and demographic variables, in the State of Pará.

Table S3: Geospatial multivariate analysis of adjusted COVID-19 rates database

Table S4**:** Data sources used in this study

Figure S2: Spatial distribution of the intersection of crude mortality, hospitalization, and fatality rates of COVID-19 in municipalities of the State of Pará, 2020-2021.

Figure S3: Spatial distribution of the intersection of adjusted mortality, hospitalization, and fatality rates of COVID-19 in municipalities of the State of Pará, 2020-2021.

Source: Authors, 2023.

Supplementary Figures

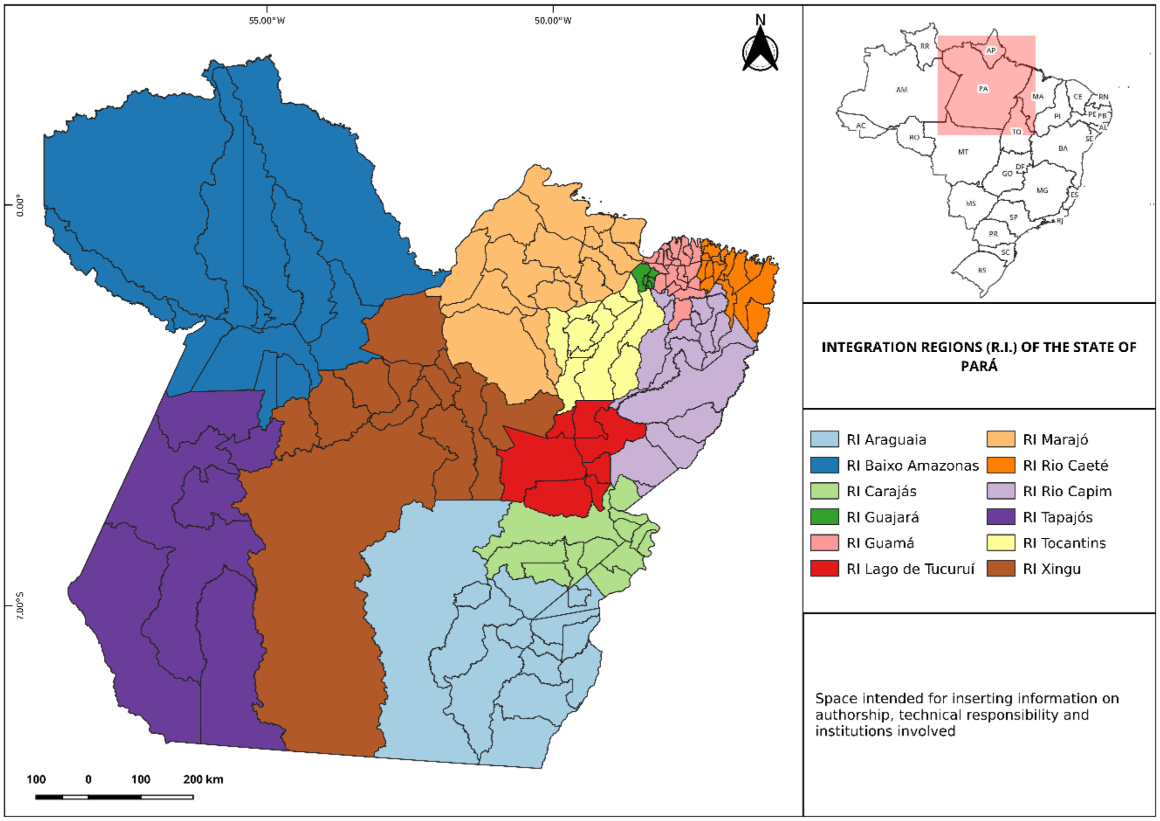

Figure S1: Map of the geographic location of the Integration Regions of the State of Pará (R.I)
Source: LEDTAM, 2023.

Supplementary Table

Table S1: Identification of municipalities according to population size classes by number of inhabitants in the State of Pará.

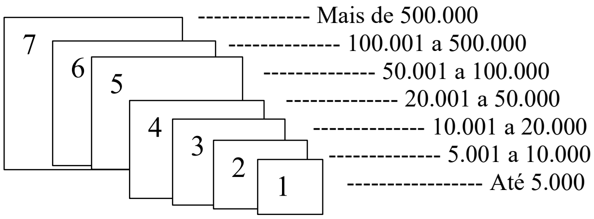

7	Belém
6	Abaetetuba, Ananindeua, Bragança, Cametá, Castanhal, Marabá, Marituba, Parauapebas, Santarém.
5	Acará, Alenquer, Altamira, Barcarena, Benevides, Breu Branco, Breves, Capanema, Capitão Poço, Dom Eliseu, Igarapé-Miri, Ipixuna do Pará, Itaituba, Itupiranga, Jacundá, Moju, Monte Alegre, Novo Repartimento, Oriximiná, Paragominas, Portel, Redenção, Santa Isabel do Pará, Santana do Araguaia, São Félix do Xingu, São Miguel do Guamá, Tailândia, Tomé-Açu, Tucuruí, Viseu.
4	Afuá, Água Azul do Norte, Almeirim, Anajás, Anapu, Augusto Corrêa, Aurora do Pará, Bagre, Baião, Bujaru, Cachoeira do Arari, Cachoeira do Piriá, Canaã dos Carajás, Chaves, Conceição do Araguaia, Concórdia do Pará, Curralinho, Curuçá, Eldorado dos Carajás, Garrafão do Norte, Goianésia do Pará, Gurupá, Igarapé-Açu, Irituia, Juruti, Limoeiro do Ajuru, Mãe do Rio, Maracanã, Marapanim, Medicilândia, Melgaço, Mocajuba, Muaná, Nova Esperança do Piriá, Novo Progresso, Óbidos, Oeiras do Pará, Ourilândia do Norte, Pacajá, Placas, Ponta de Pedras, Porto de Moz, Prainha, Rondon do Pará, Rurópolis, Salinópolis, Salvaterra, Santa Maria do Pará, Santo Antônio do Tauá, São Domingos do Araguaia, São Domingos do Capim, São Geraldo do Araguaia, São João de Pirabas, São Sebastião da Boa Vista, Soure, Tracuateua, Tucumã, Ulianópolis, Uruará, Vigia, Xinguara.
3	Aveiro, Belterra, Bom Jesus do Tocantins, Bonito, Brasil Novo, Colares, Cumaru do Norte, Curionópolis, Curuá, Floresta do Araguaia, Inhangapi, Jacareacanga, Nova Ipixuna, Nova Timboteua, Ourém, Piçarra, Primavera, Quatipuru, Rio Maria, Santa Bárbara do Pará, Santa Luzia do Pará, Santa Maria das Barreiras, São Caetano de Odivelas, São Francisco do Pará, São João do Araguaia, Senador José Porfírio, Terra Alta, Terra Santa, Trairão, Vitória do Xingu.
2	Abel Figueiredo, Brejo Grande do Araguaia, Faro, Magalhães Barata, Palestina do Pará, Pau D'Arco, Peixe-Boi, Santa Cruz do Arari, Santarém Novo, São João da Ponta, Sapucaia.
1	Bannach

*Prepared by the author based on demographic data from IBGE, Brazil 2023.
Source: Authors, 2023.

Supplementary Figures

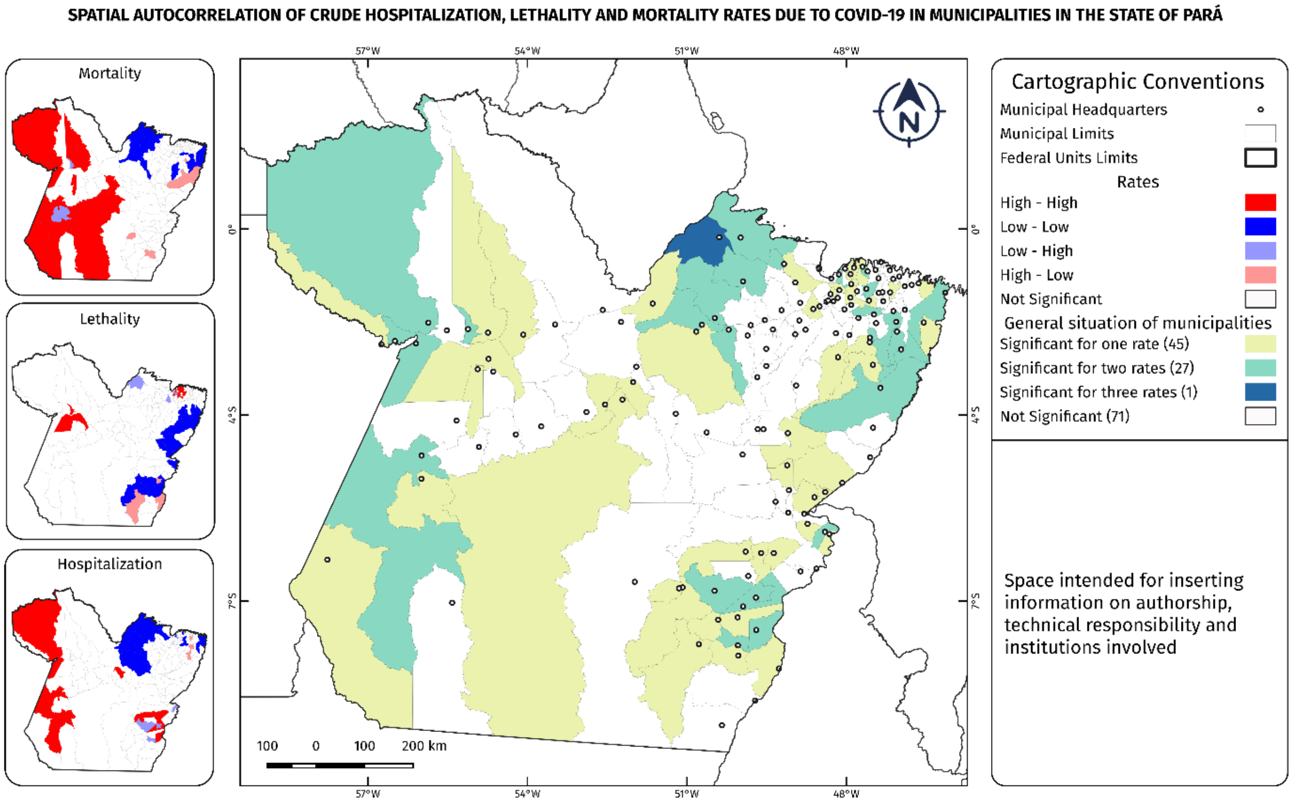

Figure S2: Spatial distribution of the intersection of crude mortality, hospitalization, and fatality rates of COVID-19 in municipalities of the State of Pará, 2020-2021.
Source: Authors, 2023.

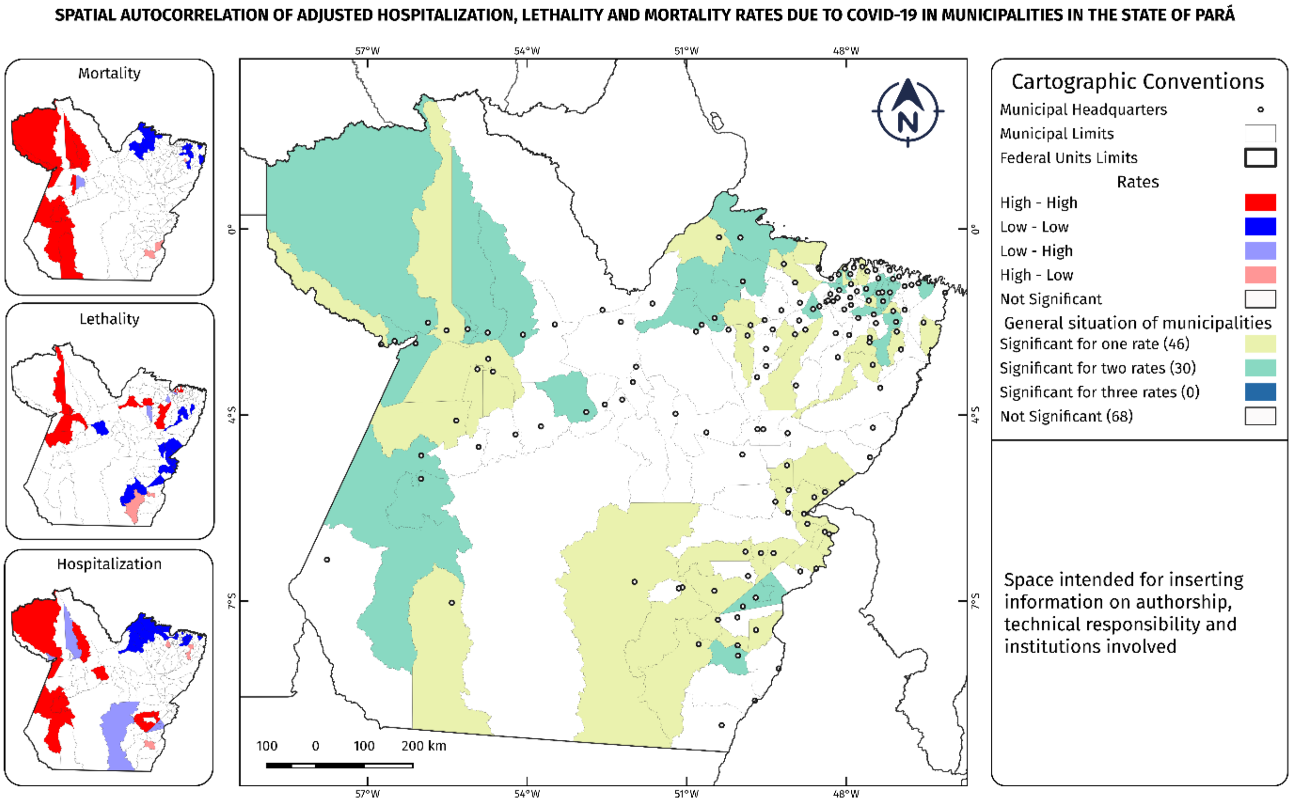

Figure S3: Spatial distribution of the intersection of adjusted mortality, hospitalization, and fatality rates of COVID-19 in municipalities of the State of Pará, 2020-2021.
Source: Authors, 2023.

Table Supplementary S2: Spearman correlation matrix of COVID-19 mortality, hospitalization and fatality rates and socioeconomic and demographic variables, in the State of Pará.

	Area	PD	GDP	Gini	HDI Income	HDI	HDI Educa	HDI Longev	ADSD	URB.D	RUR.D	%IAD	%ISD	I.D	I.URB.D	I.RUR.D
Population Density (PD)	**-0,70***															
GDP per capita	**0,37***	**-0,21***														
Gini coefficient	-0,02	**0,17***	-0,15													
HDI Income	**0,33***	0,08	**0,68***	-0,02												
Municipal HDI (HDI)	0,06	**0,31***	**0,51***	-0,00	**0,84***											
HDI Education (HDI Educa)	-0,14	**0,44***	**0,33***	-0,00	**0,62***	**0,93***										
HDI Longevity (HDI Longev)	**0,21***	-0,10	**0,46***	0,11	**0,46***	**0,47***	**0,31***									
Adequate sewage disposal (ADSD)	**-0,17***	**0,31***	**0,18***	0,00	**0,24***	**0,36***	**0,39***	**0,21***								
Urban domiciles (URB.D)	**0,17***	**0,18***	0,40	0,08	**0,70***	**0,70***	**0,60***	**0,30***	**0,27***							
Rural domiciles (RUR.D)	**-0,17***	**-0,18***	**-0,40***	-0,08	**-0,70***	**-0,70***	**-0,60***	**-0,30***	**-0,27***	**-1,00***						
% Inhabitants on adequate domiciles (%IAD)	**-0,28***	**0,39**	0,11	0,05	**0,21***	**0,38***	**0,44***	**0,18***	**0,84***	**0,29***	**-0,29***					
% Inhabitants on semi-adequate domiciles (%ISD)	0,15	0,16	0,13	-0,05	**0,35***	**0,39***	**0,36***	-0,02	-0,04	**0,24***	**-0,24***	-0,08				
% Inhabitants on inadequate domiciles (%IID)	-0,04	**-0,32***	**-0,20***	0,01	**-0,45***	**-0,54***	**-0,53***	-0,12	**-0,27***	**-0,38***	**0,38***	**-0,27***	**-0,89***			
Inhabitants per domiciles (I.D)	**-0,27***	-0,12	**-0,44***	0,09	**-0,66***	**-0,60***	**-0,48***	**-0,29***	-0,15	**-0,36***	**0,36***	-0,09	**-0,45***	**0,49***		
Inhabitants per urban domiciles (I.URB.D)	**-0,24***	-0,11	**-0,37***	0,07	**-0,59***	**-0,51***	**-0,38***	**-0,29***	-0,07	**-0,27***	**0,27***	-0,03	**-0,37***	**0,41***	**0,93***	
Inhabitants per rural domiciles (I.RUR.D)	**-0,27***	-0,10	**-0,40**	**0,14**	**-0,62***	**-0,58***	**-0,48***	**-0,25***	-0,14	**-0,33***	**0,33***	-0,06	**-0,46***	**0,48***	**0,94***	**0,82***

Source: Authors, 2023

Table Supplementary S4: Main data sources used in this study

Data source	Extracted information	Access link
1) Influenza Epidemiological Surveillance Information System (SIVEP_Gripe)<br><br>2) Pará State Health Department (SESPA).	a) Patients (date of birth, age, sex).<br>b) Residence (state, municipality, geographic area of residence).<br>c) Service (date of notification, date of hospitalization, federative unit and municipality of hospitalization).<br>d) Laboratory data (type of sample, type of antigen test, antigen test result, antigen test etiological agent, RT-PCR test result, other molecular biology method, RT-PCR etiological agent, type of serological sample for SARS-CoV-2, type of serology for SARS-CoV-2, result of serological test for SARS-CoV-2).<br>e) Conclusion of the case (final classification, case closure criteria, case evolution, date of discharge or death). From this spreadsheet, only the data of interest for the study objectives were used in the analyses.	https://opendatasus.saude.gov.br/dataset/
3) Brazilian Institute of Geography and Statistics -IBGE (Censo 2010)<br>4) Foundation for Support of Studies and Research (FAPESPA)<br>5) DATASUS	f) Demography, total population, population by urban and rural area, population by sex, population by age group, territorial area of municipalities in the State of Pará, GDP per capita, GINI coefficient, agricultural census, type of housing, number of people per domicile, adequacy of domicile, GINI coefficient by municipality.	https://censo2010.ibge.gov.br/<br>https://www.fapespa.pa.gov.br<br>https://fapespa.pa.gov.br/sistemas/anuario2020/<br>http://tabnet.datasus.gov.br/cgi/ibge/censo/cnv/ginibr.def
6) National sanitation information system (SNIS)	g) Water consumption per capita, % population with water supply	https://www.gov.br/cidades/pt-br/acesso-a-informacao/acoes-e-programas/saneamento/snis
7) Human Development Atlas in Brazil (ATLAS BRASIL)	h) Human Development index and Human Development Municipality index	http://www.atlasbrasil.org.br/
8) Mortality Information System (SIM)	i) Deaths from all causes.	Provided by Pará State Health Department (SESPA)

Source: Authors, 2023.
